# Supplementary material for: Growth performance, physiological parameters, and transcript levels of lipid metabolism-related genes in hybrid yellow catfish (Tachysurus fulvidraco ♀ × Pseudobagrus vachellii ♂) fed diets containing Siberian ginseng
Source: PLoS One. 2021 Feb 11;16(2):e0246417. doi: 10.1371/journal.pone.0246417 (PMC7877655; doi:10.1371/journal.pone.0246417)
Supplement: Attachment — Submitted filename: PLOS ONE-30481.doc [file pone.0246417.s001.doc]

PONE-D-20-30481

Growth Performance, Physiological Parameters, and Transcript Levels of Lipid

Metabolism-related Genes in Hybrid Yellow Catfish (Tachysurus fulvidraco ♀ ×

Pseudobagrus vachellii ♂) Fed with Diets Containing Acanthopanax senticosus

The present study conducted to evaluate the effect Siberian ginseng in 5levelsongrowthandphysiological performance as well as metabolism related genes in hybrid yellow catfish. Dietary levels of 2-4 g/kg showed growth promotion and reduced cholesterol and triglycerides in serum and liver. Also dietary ginseng revealed down regulation of some related gene to reduce the fat deposition in the liver.

Is this fish a kind of low fat fish which can be expressed by aquaculture activities to be high fat content? Market always prefers original taste.

The author stated that amylase, trypsin, and lipase activities in the yellow catfish intestine significantly increased with increasing of A. senticosus, but no change in FCR was found. This should be discussed appropriately.

The authors said that they measured glycogen (page 6, line 139), but no data is available in this parameter?

The present of some antinutrients in Chinese ginseng could probably affect the results and this can be considered for more discussion.

The manuscript can be accepted, but there are some minor corrections to improving the manuscript before publication.

Some of my comments are listed below:

Abstract:

Give the duration of the experiment here.

Also some numerical data should be given in summary.

Keywords:

4 of 7 exactly repeated in the title and should be moderately changed.

Introduction:

Well writing. Just some explanation on the measured parameters in this study could mature the introduction.

The culture importance and problems in the examined fish should be given.

Materials and methods:

Page 4, line 90: use the same unit for g/kg throughout the text.

Page 4, line 95-97: the preparation of the diets is not complete in terms of the amount of added water and oil, time for drying and so on.

Page 4, line 108-109: give the other water physio-chemical parameters. The tank dimension, source of water and flow rate is not given!

Page 8, line 196: the company info for this version of SPSS in not correct.

Results:

Well writing.

Give the final weight of fish for better comparison by readers.

Discussion:

Page 13, line 292: change rate to level.

Page 13, line 296-298: but no changes were observed in FCR.

Page 13, line 299: should be present as strongest effect on…

Page 13, line 300-302: not related to the changes of enzymes. Some ingredients and the levels should be an effective reason.

Page 13, line 306-308: but the authors did not measure the catabolic enzymes.

Page 13, line 309-310: this sentence is repeated in introduction.

Page 14, line 336: change to HSI.

Page 15, line 369-370: I'm not agree with this as those are antinutrients and many studies showed the negative impacts of these on growth and metabolism of animals.

Reference:

There are many mistakes in references in terms of format, journal abbreviations, scientific names, etc. such as lines 394-395, 411,414-415,417, 421-422, and so on.

Tables:

Table 1: give the unit in parenthesis in front of the ginseng. Correct middling with small letter. Put all proximate analyses of the diets in terms of ash, dry matter and other items.

Table 3: insert the initial and final fish weights.

Table 4: why no data on T3, T3 and the ratio of T3:T4 are available in this table!? Those should be given here. The unit of TP is generally g/dL.

Figures:

The quality of all figures is low in the reviewers' file.

Figure 1: no data are available to how the broken-line was analyzed.

Figure 2: change value to ratio.
